# Hyperspectral and multispectral image processing for gross-level tumor detection in skin lesions: a systematic review

**DOI:** 10.1117/1.JBO.27.6.060901

**Published:** 2022-06-08

**Authors:** Eleni Aloupogianni, Masahiro Ishikawa, Naoki Kobayashi, Takashi Obi

**Affiliations:** a Tokyo Institute of Technology, Department of Information and Communication Engineering, Tokyo, Japan; b Saitama Medical University, Faculty of Health and Medical Care, Saitama, Japan; c Institute of Innovative Research, Tokyo Institute of Technology, Yokohama, Japan

**Keywords:** gross pathology, hyperspectral, classification, skin lesions, medical image processing

## Abstract

**Significance:**

Skin cancer is one of the most prevalent cancers worldwide. In the advent of medical digitization and telepathology, hyper/multispectral imaging (HMSI) allows for noninvasive, nonionizing tissue evaluation at a macroscopic level.

**Aim:**

We aim to summarize proposed frameworks and recent trends in HMSI-based classification and segmentation of gross-level skin tissue.

**Approach:**

A systematic review was performed, targeting HMSI-based systems for the classification and segmentation of skin lesions during gross pathology, including melanoma, pigmented lesions, and bruises. The review adhered to the 2020 Preferred Reporting Items for Systematic reviews and Meta-Analyses (PRISMA) guidelines. For eligible reports published from 2010 to 2020, trends in HMSI acquisition, preprocessing, and analysis were identified.

**Results:**

HMSI-based frameworks for skin tissue classification and segmentation vary greatly. Most reports implemented simple image processing or machine learning, due to small training datasets. Methodologies were evaluated on heavily curated datasets, with the majority targeting melanoma detection. The choice of preprocessing scheme influenced the performance of the system. Some form of dimension reduction is commonly applied to avoid redundancies that are inherent in HMSI systems.

**Conclusions:**

To use HMSI for tumor margin detection in practice, the focus of system evaluation should shift toward the explainability and robustness of the decision-making process.

## 1 Introduction

More people are diagnosed with skin cancer each year in the United States than all other cancers combined.^1^ Malignant melanoma (MM) and other skin cancer crude mortality rates increased up to twofold in Japan from 1999 to 2014, independent of the aging of the Japanese population.^2^ The increased workload demands increased effectiveness in the diagnosis and management of malignant lesions. Skin lesions of concern are evaluated clinically and/or histopathologically. Gross pathology, the intermediate stage between the two, remains a manual, labor-intensive, and non-standardized step in the diagnostic work flow,^3^ despite recent advances in digital pathology.^4^ Hyperspectral imaging (HSI) is an imaging modality that can assist gross pathology. HSI captures spectral signatures of tissue pixels dependent on substance concentrations inside the tissue and has been proven useful for staining and standardization in histopathology.^5^ In the past decade, several research groups have investigated applications of HSI and multispectral imaging (MSI) at the gross pathology stage, toward diagnosis and tumor classification of skin lesions, aiming to achieve noninvasive optical biopsies and increase diagnosis speed.

### 1.1 Obstacles in Pathology

There is an inherent compromise in skin cancer diagnosis, to both not miss cases in the early stage and to avoid unnecessary excisions. The gold standard is a histopathological biopsy, which requires several days to produce results. Although the clinical setting differs between primary and secondary care, the compromise persists. Grossing is performed by an experienced anatomic pathologist, who evaluates the excised tissue specimen before the microscope evaluation. The purpose of anatomic pathology is to accurately determine tumor margins. Early detection *in situ*, namely before grossing, improves considerably the disease prognosis, especially for melanoma cancers. The current state-of-the-art in clinical practice is dermoscopy, which can improve the diagnosis of skin cancer, but still suffers in terms of specificity.^6^ Gross pathology lacks automation and cross-laboratory protocol standardization, which introduces discrepancies in diagnosis. In addition, it results in over-reliance on the skill of the pathologist. Therefore, patient and medical staff must wait for the biopsy, which apart from inducing costs due to the delay increases the probability of resection due to the unavailability of tumor margins pre- and intraoperatively. The discovery of compromised cancer margins results in increased overall treatment costs, treatment duration, and patient discomfort increase. Along with the increased prevalence of skin cancer^1^^,^^2^ and the shortage of experienced pathologists, a substantial workload is assigned to the pathology lab. Furthermore, amid the global Covid-19 pandemic, enhancing the digital flow and offering opportunities for remote education and diagnosis are quickly becoming the turning point of medical research.

### 1.2 Optical Properties of the Skin

Optical properties of the tissue are commonly used in diagnostic systems. Considering the reflectance model, an incident illumination ray is partially refracted inside the target and partially reflected. Reflectance is a function of wavelength, modified by the target tissue in accordance to Beer–Lambert Law of absorbance.^7^ Light rays scatter heavily inside human skin, which is structured in layers.^8^ Carcinogenesis and associated metabolic changes, known as Warbung effect,^9^ modify the molecular structure of the affected tissue, altering the distribution of chromophores. These heterogeneous changes affect the ratio of absorbed and reflected light. Thus, the reflectance spectrum can be regarded as a descriptive signature of the chromophore contents of the tissue, analogous to the human fingerprint. Consequently, discordant signatures can describe atypical concentrations of skin chromophores and be an indicator of abnormal growths.^10^ HSI or MSI systems are one of the means to record such signatures.

### 1.3 Spectral Imaging

A range of spectral imaging technologies have been developed, owing to its noninvasive nature.^11^ Raman spectroscopy (RS) uses fiber optic probes to capture information about the molecular fingerprint of a tissue. Although applications of RS in macrolevel diagnosis have been attempted, the complexity of the imaging system design and the need for guidance remains an obstacle.^12^ Another approach on *ex vivo* gross samples is multispectral Mueller polarimetry imaging,^13^ a complex laser-based technique. When depth-imaging is the focus, multispectral optoacoustic tomography can be employed. This label-free technique uses fast laser pulses that excite the tissue, producing waves that can be reconstructed using backpropagation.^14^ The main drawback of those methods in a gross-level application is the increased component complexity, need for customization, limited field of view, and bulky equipment. In addition, the aforementioned methods are point-based, which impedes wide-area snapshots and imaging duration. Alternatively, spectral imaging equipment can be used in combination with fluorescent agents, to provide labeled images of the tissue. Despite the labeling advantage, this approach suffers from poor spatial resolution and poor tissue mapping due to movement.

### 1.4 Hyper/Multispectral Imaging

HSI was originally developed for remote sensing and space applications. However, HSI and MSI are emerging imaging modalities for medical applications, as they can capture the tissue’s spectral signature. RGB cameras mimic the behavior of cone cells in the human eye, showing three distinct wide-band responses to visible light. S-cells integrate radiation information in the range 420 to 440 nm, M-cells in 535 to 545 nm, and L-cells in 564 to 580 nm. RGB cameras have similar wide-band integration filters, therefore, are susceptible to metamerism, i.e., the inability to recognize different colors under a certain illumination.^15^ In contrast, HSI and MSI use narrow-band filters with a width of a few nm. The spectral range can be either at the visible wavelengths (VIS, 380 to 780 nm) or the near-infrared range (NIR, 780 to 2500 nm). Longer wavelengths offer the additional ability to penetrate through the deeper layers of the skin. Depending on the imaging equipment, it is possible to capture a two-dimensional (2D) surface instantly, with good spatial accuracy. HSI and MSI differ in terms of the number of channels. MSI systems are usually customized to the absorbance features of the target tissue. As a result, an MSI pixel describes essentially a feature vector, in contrast to the smooth HSI reflectance curves. In this study, we will consider images with ≤20 channels as MSI, while the rest will be considered HSI.^16^ We refer to both techniques jointly as hyper/multispectral imaging (HMSI).

### 1.5 Potential in Diagnosis

The application of HMSI in pathology shows significant potential.^17^ First, it is a noncontact, noninvasive, nonionizing imaging method. HMSI does not modify the physical (cellular- and tissue-level) properties of the tissue while preserving the spatial dimension of the distribution of tissue chromophores. Second, HMSI is fast compared with histopathology. An image can be acquired and processed in a few seconds or minutes instead of days. Coupled with a semi- or fully automated processing tool, HMSI-based tumor segmentation can be implemented easily and with minimal training of the medical personnel. In turn, associated costs can be reduced, and resources can be reserved for the diagnosis of more difficult cases. Skin tissue, which is characterized of an increased presence of chromophores and surface inconsistencies, is an ideal target for colorimetric and texture analysis. Technological advances in hardware, GPU programming, and machine learning libraries facilitate the processing of HMSI data, which often require a few GB of memory per image. HMSI has been investigated for applications in tumor detection, dermoscopy,^18^ and temporal monitoring.^19^ Previous studies on commercial MSI-based diagnostic tools showed a considerable increase in sensitivity and specificity for histology guidance by dermatologists and non-dermatologist clinicians.^20^^,^^21^ Several studies report trends in applications^22^ and classifications schemes for HMSI.^23^ Specifically for medical applications, previous reviews focused on noninvasive methods,^24^ comparison of HMSI to RGB images and dermoscopy,^25^ and commercial systems^26^^,^^27^ for *in situ* melanoma detection, with mixed findings. However, as of writing this report, we are not aware of any systematic review that investigates HMSI for gross pathology for different types of skin lesions, including nonmelanoma cancers.

### 1.6 Objectives

HMSI has the potential to standardize, accelerate, and facilitate diagnosis, by (a) producing tumor segmentation in the form of optical biopsies (intraoperatively) or by (b) clarifying cancer margins at the clinical stage (preoperatively). An HMSI imaging system combined with a powerful processing algorithm would reduce diagnostic costs, due to the lack of moving parts, fast acquisition, robustness, and application on multiple pathologies. Through this systematic review, we expect to identify current trends in data processing for HMSI-based segmentation schemes for skin lesions at the macroscopic level. In addition, we summarize HMSI acquisition systems and preprocessing techniques, as well as study effects and limitations.

This study is structured as follows. In Sec. 2, we describe the methodology for this systematic review. In Secs. 3–5, we explain in detail proposed schemes for data acquisition, preprocessing, and classification/segmentation, respectively, show cumulative results from the review and explain subcategories. In Sec. 6, we discuss effects, drawbacks, and common limitations of HMSI processing for skin lesions. The final conclusions are summarized in the last section.

## 2 Materials and Methods

### 2.1 Characteristics of the Skin

**Fig. 1:**
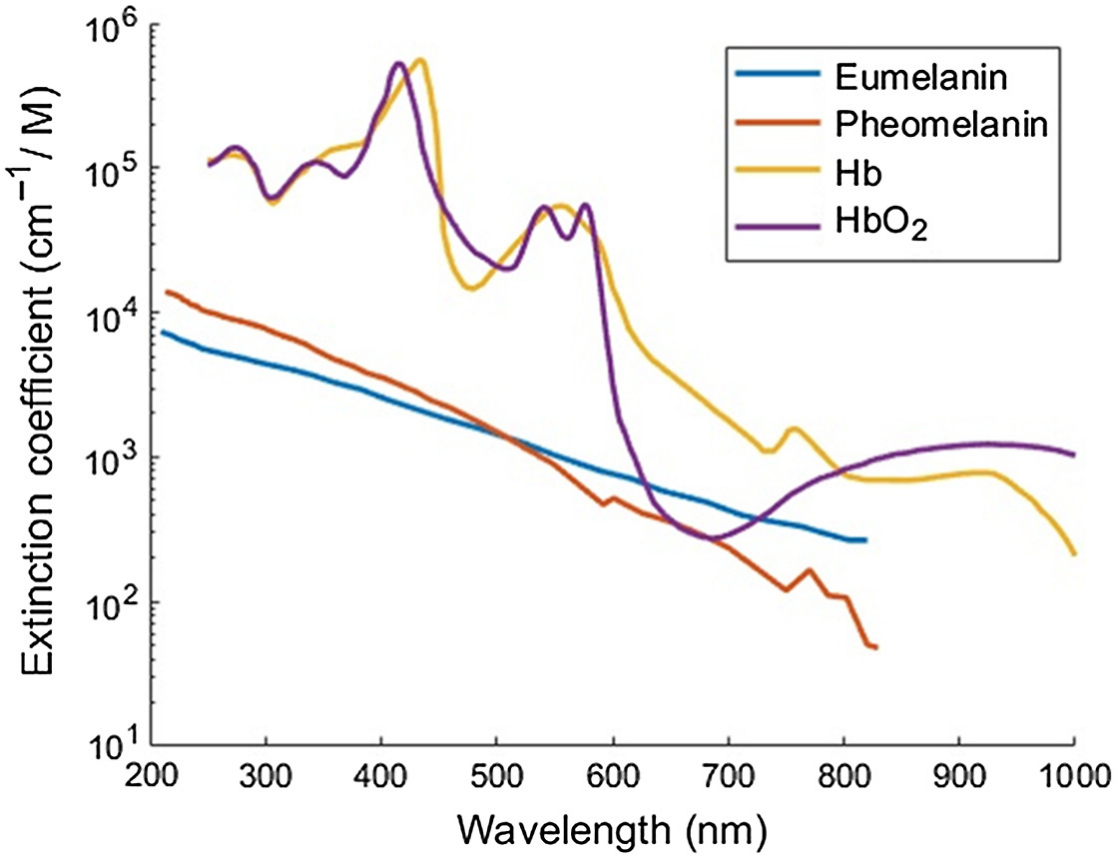
Spectra of the extinction coefficient for the three main chromophores of skin tissue. Reconstruction using data from Ref. 28.

The optical signature of a tissue is characterized by the concentrations of chromophores inside the tissue. The main chromophores of the skin are melanin (eumelanin and pheomelanin), oxygenated hemoglobin (${\mathrm{HbO}}_{2}$), and deoxygenated hemoglobin (Hb), with strong absorbance in the VIS range. Lipids and water have strong absorbance in the NIR. Absorbance is proportional to the extinction coefficient for fixed concentration and optical path, according to the Beer–Lambert Law. The extinction coefficient spectra of skin chromophores are shown in Fig. 1. Response to characteristic peaks and valleys of these curves is routinely used in the development of HMSI systems.

Human skin is an organ comprised of tissue layers consisting of three different types of cells, namely squamous, basal, and melanocytic cells. Malignancies appear when skin cells start to multiply uncontrollably. Although each skin cell category is responsible for different types of skin cancer, irregular growth of melanocytes is the cause of melanoma. Melanoma is the most aggressive skin cancer and is associated with most skin cancer-related deaths. Occasionally, irregular cell growth can be benign, with the example of nevi and Spitz nevi, which are nonthreatening melanocytic tumors. Therefore, discrimination between benign and malignant melanocytic lesions is difficult due to the similar cellular substrate. A common category for HMSI-related research is pigmented skin lesions (PSL), because of the enhanced and heterogeneous presence of skin chromophores. Pigmented skin lesions include MM, basal cell carcinoma (BCC), and squamous cell carcinoma. Another category rich in color information is injury lesions, bruises, and burns.

Knowledge about spectral properties of skin tissue can assist the development of diagnostic models. A high rate of vascularization combined with a high amount of melanocytic cells, high hemoglobin, and melanin concentrations, respectively, can indicate the presence of malignancy. ${\mathrm{HbO}}_{2}$ and Hb show absorbance peaks around 430 nm, while ${\mathrm{HbO}}_{2}$ has a local minimum of absorbance at 470 nm. ${\mathrm{HbO}}_{2}$ shows twin peak absorbance at 550 and 570 nm and Hb a local peak at the same area. At 660 nm absorbance of ${\mathrm{HbO}}_{2}$ drops suddenly. After 730 nm, the absorbance of melanin starts to become prominent and ${\mathrm{HbO}}_{2}$ can be discriminated from Hb. Spectral bands at 950 nm provide information from the deeper layers of the skin. Borisova et al.^29^ and Zherdeva et al.^30^ showed that the reflectance spectra of pigmented MM above 600 nm are consistently lower than that of nevi or other pigmented lesions. Healthy tissue has consistently higher reflectance compared with lesion tissue. Healthy skin and pigmented lesions rich in blood content show a sudden reflectance hike at 570 nm, although reaching a different maximum reflectance value.

### 2.2 HMSI Imaging

HMSI describes jointly HMSI systems. Some studies do not discriminate between HSI and MSI due to their similar nature. However, some notable differences are present. Due to obstacles in technology, initial remote sensing systems were capable only of MSI.^16^ MSI systems sample only specific wavelengths or wavelength bands. Therefore, one can select individual wavelengths with classification capabilities and record only their specific reflectance values, resulting in a small number of channels. As technology advanced, HSI systems became feasible. HSI systems sample the entire spectrum with a fixed step and provide continuous spectral signatures. In problems where target wavelengths are unknown, the continuous spectral signature might reveal a spectral pattern that is concealed in the MSI. On the other hand, the increase in the number of channels adds additional complexity in data storage and processing, as well as increases processing time.

HMSI for pathology-related tasks is usually performed in the VIS range, where chromophore absorbance is highest. The NIR range is useful when detailed information for depth structures is required because longer wavelengths can penetrate deeper. However, the spatial quality of NIR tends to be reduced, due to increased scattering and attenuation phenomena. The quality of HMSI differs between *in situ* and *ex vivo* imaging, due to the influence of breathing and patient’s movement.

### 2.3 Review Methodology

In this systematic review, we followed the methodology proposed in the updated 2020 Preferred Reporting Items for Systematic reviews and Meta-Analyses (PRISMA) statement.^31^

#### 2.3.1 Eligibility criteria

The goal of the review is to investigate the current status and trends regarding applications of MSI or HSI of skin tissues for the tasks of segmentation and classification at a macroscopic level. Imaging range is limited to VIS and NIR ranges, while datasets consist either of *in situ* or fresh *ex vivo* (<3  h after excision) gross samples from humans and/or mammals. Studies using fluorescence and tomography are out of the scope of this review. The search for eligible manuscripts was conducted among full papers (journal, conference, and technical reports) written in English and published from 2010 to 2020.

#### 2.3.2 Information sources

The digital libraries of Scopus and PubMed were used as information sources. Both search engines are free and index a comprehensive catalog of recent publications in the fields of life science and biomedical engineering. Database search and result retrieval were performed on October 20, 2021.

#### 2.3.3 Search strategy

The search condition is described in Eq. 1. The keyword search was limited to Title and Abstract, and not full body, to avoid retrieving manuscripts with keyword mentions in passing. A list of result entries was retrieved and processed for duplicate entry identification:

C=(Pathology—Gross Pathology—Macropathology)&(Hyperspectral—Hyper-Spectral—Multi-Spectral—Multispectral)&(Language=English)&(PubYear≥2010)&(PubYear≤2020). (1)

#### 2.3.4 Selection process

A researcher (EA) performed initial screening of search results, using Title and Abstract. Result items that linked to single-page or non-English reports were discarded. Eligibility criteria were applied liberally during the initial screening. Reports that passed the initial screening were retrieved and screened strictly for eligibility.

#### 2.3.5 Data collection process

For each eligible report, a researcher (EA) summarized contents and extracted relevant data, which were examined by every researcher. All eligible studies were cross-referenced for updates, errata, and retractions.

#### 2.3.6 Data items

Eligible outcomes were categorized broadly according to tissue state (*in situ* or *ex vivo*) and classification task. Outcomes from studies that included small datasets or no measured outcome were reduced in importance but were not excluded. The following data items were collected for each report; publication details (author, year), study design (imaging equipment, preprocessing, processing framework, ground truth domain), dataset characteristics (number of subjects, target lesions, *in situ*, or *ex vivo*), and performance (accuracy metrics, benefits, limitations).

#### 2.3.7 Risk of bias

Selection of eligible studies was performed by one researcher (EA). To reduce bias in the selection process, the search was performed in two steps. Each candidate report was screened twice, with liberal and strict eligibility criteria. In addition, we searched two nonexclusive databases, increasing the chances of coming across a candidate study. Finally, we reviewed relevant reports that were references of or citations to eligible reports, as well as high impact reports, defined as having more than 50 citations on Google Scholar.

#### 2.3.8 Effect measures

In pathology, there is an inherent compromise between not missing any dangerous lesion (high sensitivity) and not rushing patients to unnecessary treatment due to false positives (high specificity). The balance between the two depends on the targets defined by the clinic. Dermoscopy evaluation is the state-of-the-art for clinical evaluation. Histology-validated tumor margins remain the golden standard for skin lesion diagnosis.

The effects of each eligible study were compared using accuracy (Acc.), sensitivity (Sens.), and specificity (Spec.) metrics. The metrics are calculated as

$$\mathrm{Acc}.=\frac{\text{TruePositives}+\text{TrueNegatives}}{\text{Total Number of Samples}},$$ (2)

$$\mathrm{Sens.}=\frac{\text{TruePositives}}{\text{TruePositives}+\text{FalseNegatives}},$$ (3)

and

$$\mathrm{Spec}.=\frac{\text{TrueNegatives}}{\text{TrueNegatives}+\text{FalsePositives}},$$ (4)

where true/false indicates whether the predicted value is true/false compared with the ground truth. The importance of effects was adjusted according to dataset size (number of lesions).

Depending on the task, different types of ground truth can be used. Image-wide labels can be either a binary classification (malignant versus benign) or the disease name. Alternatively, the image can be split into patches, each with its patch-wide label. Finally, pixelwise labeling is a segmentation mask of disease/tumor pixels. The labels are usually created by medical personnel either (a) after the clinical examination using drawing tools, (b) by registration and mapping of gross pathology data to histology data, or (c) by labeling gross tissue itself using a fluorescent agent. The type of ground truth (dermoscopy, histology, or other) was reported, but not factored into evaluation.

#### 2.3.9 Synthesis methods

Due to the heterogeneity of datasets, imaging equipment, and outcome measures, a meta-analysis could not be performed. Instead, we performed a narrative synthesis describing performance in different task categories and provided comparative plots and tables of performance metrics.

#### 2.3.10 Reporting bias assessment

It should be noted that both screened and eligible reports may be affected by publication bias, as studies with nonsignificant results of HMSI applications did not reach the publication stage. In addition, some results, for example, comparisons to the golden standard or alternative methods, may be omitted from the studies due to selective reporting bias.^32^

#### 2.3.11 Certainty assessment

For each study, sample size and methodology limitations were used for the certainty assessment of reported effects. In cases where fidelity estimates or feedback from medical personnel were reported, these were taken into account.

#### 2.3.12 Study selection

**Fig. 2:**
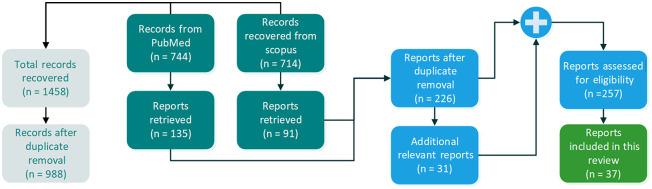
Flowchart of publication selection for this systematic review.

The flowchart of publication search and selection is described in Fig. 2. The search returned 744 entries from Scopus and 714 entries from PubMed. Afterward, we screened for eligibility (a) reports that cited to and (b) reports that were cited by these initial reports. A total of 37 reports fulfilled the eligibility criteria. A list of all eligible studies and additional information (sample size, channel number, spectral range, validation labels, and tissue type) is provided in Table 1.

**Table 1:** List of eligible studies.

Ref.	Range (nm)	Channels	Dataset size	Validation	Tissue
33	400 to 1000	162	23 subjects	hist.	*In situ*
34	405 to 970	19	96 lesions	derm.	*In situ*
35	VIS-NIR	N/A	1211 lesions	mult.	*In situ*
36	N/A	10	1632 lesions	derm.	*In situ*
37	450 to 950	50	81 lesions	hist.	*In situ*
38	450 to 950	50	82 lesions	derm.	*In situ*
39	380 to 780	124	24 subjects	hist.	*In situ*
40	380 to 780	124	27 subjects	hist.	*In situ*
41	400 to 1100	71	26 lesions	hist.	*In situ*
42	380 to 780	124	20 subjects	hist.	*In situ*
43	540 to 950	3	3 lesions	derm.	*In situ*
44	1000 to 2400	140	1 subject	other	*In situ*
45	360 to 1000	1127	64 subjects	mult.	*In situ*
46	500 to 850	71	52 lesions	derm.	*In situ*
47	450 to 990	55	32 lesions	hist.	*In situ*
48	380 to 780	124	80 subjects	hist.	*In situ*
49	500 to 850	71	19 subjects	derm.	*In situ*
50	400 to 982	8	18 lesions	hist.	*In situ*
20	430 to 950	10	6 lesions	derm.	*In situ*
51	500 to 885	76	8 lesions	hist.	*In situ*
52	450 to 750	61	4 lesions	N/A	Both
30	450 to 750	61	45 subjects	hist.	*In situ*
53	540 to 950	51	126 lesions	derm.	*In situ*
54	500 to 850	71	32 lesions	hist.	*In situ*
55	400 to 720	33	26 subjects	hist.	*In situ*
56	N/A	10	360 lesions	hist.	*In situ*
57	N/A	N/A	N/A	N/A	N/A
58	414 to 995	8	429 lesions	derm.	*In situ*
59	450 to 950	50	124 lesions	N/A	*In situ*
29	345 to 1040	N/A	N/A	N/A	*Ex vivo*
60	500 to 850	71	16 lesions	hist.	*In situ*
61	450 to 630	7	10 subjects	derm.	*Ex vivo*
62	450 to 750	125	49 lesions	derm.	*In situ*
63	450 to 780	34	4 subjects	derm.	*In situ*
64	450 to 750	125	76 lesions	derm.	*In situ*
65	398 to 757	84	619 lesions	hist.	*In situ*
66	398 to 757	84	619 lesions	hist.	*In situ*

Note: N/A, not available; derm., dermatology,; hist., histology,; mult., multidisciplinary. Items are ordered according to publication year.

## 3 Data Acquisition

**Fig. 3:**
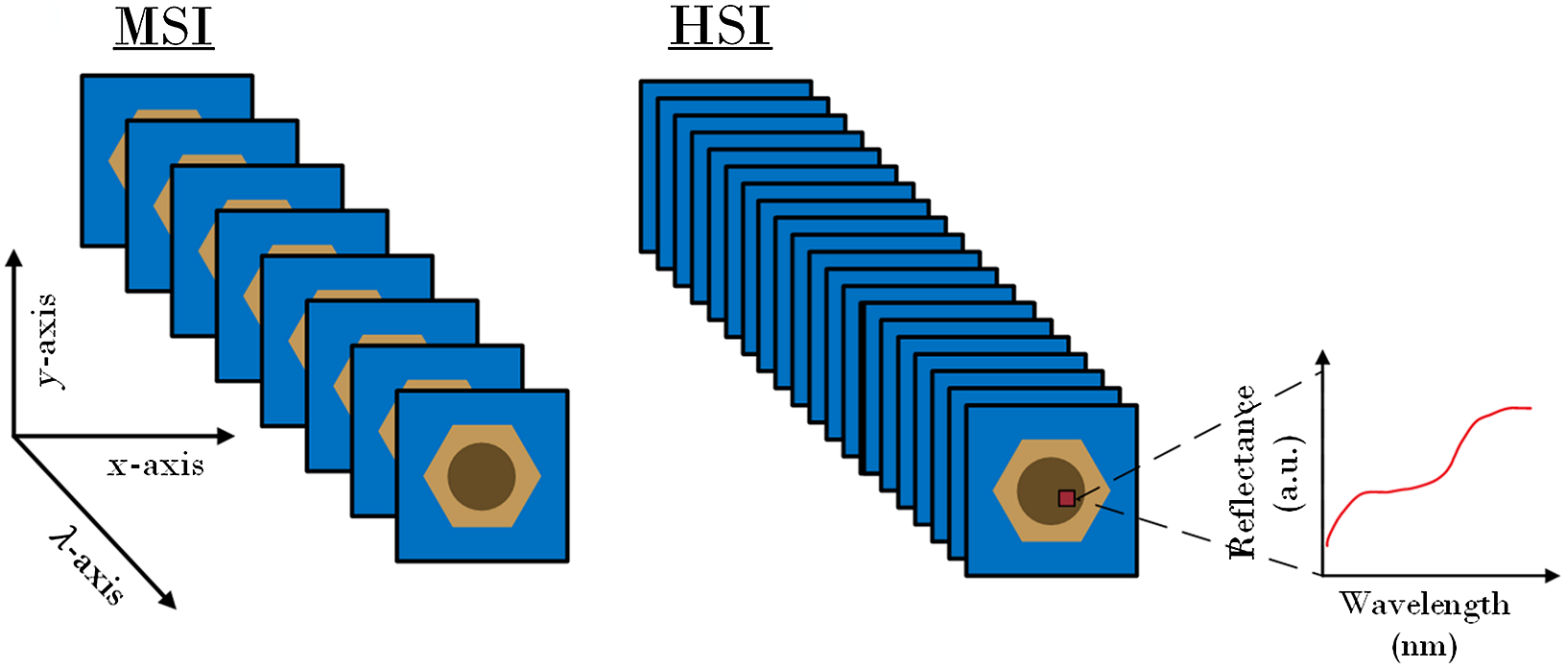
Example of the structure of acquired MSI (left) and HSI (right) data cubes.

HMSI systems capture the reflectance spectrum of a surface, where each pixel’s information represents the spectral signature of the tissue at that location. HMSI cameras can be assigned into four broad categories, depending on the scanning function used to obtain the image cube. These are: (a) whiskbroom or point-scanning, cameras, (b) pushbroom or line-scanning, cameras, (c) cameras based on spectral scanning (area-scanning or plane-scanning), and (d) snapshot (single shot) cameras. The HMSI image cube has two spatial (x,y) and one spectral (λ) dimension, which can be viewed as a stack of spectral subimages, as shown in Fig. 3. Each pixel value of a subimage can be expressed as the integrated product of reflectance R, camera sensitivity S, and illumination E spectra. This can be described as

$$I_{i}(x,y)=\int_{\lambda_{\min}}^{\lambda_{\max}}S(x,y,\lambda )E(x,y,\lambda )R(x,y,\lambda )d\lambda +n_{i},$$ (5)

where $I_{i}$ is the image pixel at the i’th channel, $n_{i}$ is the noise at the i’th channel, x, y are the spatial dimension variables, λ is the spectral dimension variable, and $\lambda_{\min}$, $\lambda_{\max}$ are the narrow-band filter limits. For biological tissues, reflectance corresponds to diffuse reflectance spectra, after phenomena of absorbance and scattering take place.

**Fig. 4:**
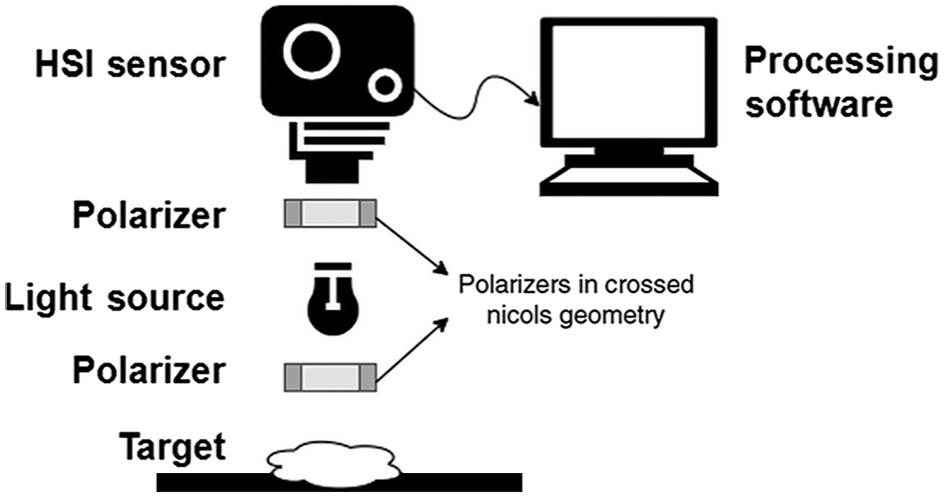
The core components of an HMSI system. It includes a camera sensor, a light source, and two polarizers in cross Nicols geometry (one in front of the camera and one in front of the light source). To accommodate this schematic, the light source can be mounted on a ring part.

The core components of a typical HMSI system are shown in Fig. 4. The sensor is positioned above the target, same as the light source. To achieve both, the LED can be attached on a ring part. Otherwise, the light source can be positioned at the side, at a 45-deg angle to the capture base. A pair of polarizers are positioned in front of the sensor and in front of the light source, to remove saturation and isolate information from the tissue surface. The sensor is usually connected to a compute with software for imaging control. The details of systems used in the literature are presented in Tables 2 and 3, for HSI and MSI, respectively.

**Table 2:** Overview of HSI systems.

Range (nm)	Step (nm)	Res. (px)	Density	Area	Duration	Port.
VNIR 1600 (Norsk Elektro Optikk AS, Norway)^33^						
Push-broom scanning. Distance 1 m from target surface.						
400 to 1000	3.7	N/A	N/A	N/A	N/A	No
N/A (Galderma R&D, Switzerland)^34^						
405 to 970	25	900×1200	N/A	N/A	N/A	N/A
SIAscope V (MedX Health, Canada))^35^						
Commercial system in circulation. End-to-end imaging to diagnosis.						
N/A	N/A	N/A	N/A	N/A	∼sec	Yes
Nuance EX (CRi, USA)^38^^,^^44^^,^^53^^,^^59^^,^^67^						
450 to 950	10	N/A	0.05 mm/px	N/A	2 to 5 min	No
Custom MSI-2 (Mitaka Kohki, Japan)^39^^,^^40^^,^^42^^,^^65^^,^^66^						
Line scanning.						
380 to 780	2.4	512×512	13 μm/px	$36\;{\mathrm{mm}}^{2}$	20 s	No
Custom (2×Mono CCD+tunable filter)^41^^,^^47^						
Distance 20 cm from target surface.						
650 to 1100	10	N/A	N/A	$64\;{\mathrm{cm}}^{2}$	N/A	Yes
SWIR (Specim, Finland)^44^						
Line scanning. Target on movable tray.						
1000 to 2400	10	320×N/A	0.2 mm/px	N/A	N/A	No
AvaSpec 2048 (Avantes, Netherlands)^45^						
Optical fiber connected to probe at 5 mm from target surface.						
360 to 1000	0.6 to 0.53	N/A	N/A	$60\;{\mathrm{mm}}^{2}$	N/A	N/A
FPI VIS-VNIR (VTT, Finland)^46^^,^^49^^,^^51^^,^^60^						
Snapshot scanning. Distance 4 cm from target surface.						
500 to 850	5 to 10	320×240	N/A	$12\;{\mathrm{cm}}^{2}$	∼sec	Yes
Custom (digital camera+tunable monochromator)^30^^,^^52^						
Overhead camera away from the target surface.						
450 to 750	5	1360×1024	N/A	$49\;{\mathrm{cm}}^{2}$	N/A	No
Custom (Mono CCD+tunable filter)^55^						
Overhead camera away from the target surface.						
400 to 720	10	N/A	N/A	N/A	4 min	No
USB4000 (Ocean Optics, USA)^29^						
345 to 1040	1.5 to 2.3	N/A	N/A	N/A	N/A	N/A
UHD 185 (Cubert GmbH, Germany)^62^^,^^64^						
Attached directly on the target surface with contact ring.						
450 to 750	8	50×50	N/A	$144\;{\mathrm{mm}}^{2}$	250 ms	Yes
IMPULSO (PUCP, Peru)^63^						
450 to 780	10	1312×1082	N/A	$12.16\;{\mathrm{cm}}^{2}$	N/A	N/A

Note: N/A, not available; Res., resolution; density, pixel density; area, capture area; port., portability.

**Table 3:** Overview of MSI systems.

Range (nm)	Channels	Res. (px)	Density	Area	Duration	Port.
MelaFind (MELA Sciences, SUA)^20^^,^^36^^,^^56^						
Commercial system, discontinued. End-to-end imaging to diagnosis.						
430 to 950	10	N/A	N/A	N/A	N/A	No
Custom (monochromatic camera+bandpass filter)^50^						
Sequential capturing at each filter.						
VIS	8	N/A	N/A	N/A	N/A	N/A
Custom (monochromatic CCD+8 LED)^58^						
Sequential capturing at each LED.						
414 to 995	8	1280×960	N/A	$15\times 20\;{\mathrm{mm}}^{2}$	40 s	Yes
Custom (IDS camera+4 LED)^58^						
Sequential capturing at each LED.						
VIS-NIR	4	1280×960	N/A	$30\times 30\;{\mathrm{mm}}^{2}$	40 s	Yes
Custom (RGB camera+7 LED)^61^						
Sequential capturing at each LED.						
450 to 630	9	2320×1735	N/A	N/A	∼min	No

Note: N/A; not available; Res.; resolution; density, pixel density; area, capture area; port., portability.

### 3.1 Commercial Systems

A relatively fast approach for studies that investigate proof-of-concept is the use of commercial HSI systems. Four studies used commercially available HSI^35^ and MSI systems^36^^,^^20^^,^^56^ specifically designed for skin diagnosis. MelaFind, a commercial tool for melanoma detection, which uses 10 MSI channels (430, 470, 500, 550, 600, 650, 700, 770, 880, and 950 nm), showed an increase in specificity and sensitivity compared with dermoscopic/clinical diagnosis.^36^ SIAscope, another commercial solution, captures spectral information in both the VIS and NIR range.^35^ Several reports used the Nuance EX system coupled with additional halogen lamps and a polarizing film on the lens^67^^,^^38^^,^^59^ or with a single halogen lamp at 45 deg/0 deg geometry.^44^ Nishino et al.^44^ used the VIS HSI only for visualization purposes, while simultaneously using an NIR line-scan camera for data collection. Randeberg et al.^33^ used the VNIR camera and illuminated the scene with two halogen lamps, with sandblasting to reduce specular reflectance. Liu et al.^45^ combined the HSI camera with a halogen source and a reflection fiber optics probe, adjusted to achieve illumination at 45 deg.

### 3.2 Hyperspectral Prototype Systems

Some of the reports made use of immobile imaging structures, created specifically for their use case. Nagaoka et al.^39^ used a prototype HSI system that combined an imaging spectrograph with an electron multiplying charge-coupled device camera. The system included a halogen lamp with optic fiber, a cylindrical lens, and two polarizers. They used a frame rate of 30  frames/s to time line-scanning. Kim et al.^55^ created a prototype HSI system using a monochromatic charge-coupled device (CCD) camera and a liquid crystal tunable filter. A telecentric lens with 0.3× magnification was mounted on the camera. Diffuse illumination was provided through a ring light. Suárez et al.^41^ used two different monochromatic CCD cameras and tunable filters to capture spectra in the VIS and NIR ranges. The cameras had different resolutions, so they were registered by mutual information maximization. Carmona Jaramillo et al.^63^ analyzed images from research prototype IMPULSO, but did not provide additional information regarding system design. Zherdeva et al.^30^ used an experimental setup comprised of a tunable monochromator and broadband LED. The filtering at each wavelength was achieved using a controller-operated acoustic wave generator combined with two amplifiers. Two polarizers are also included in the design.

Apart from immobile systems, a few handheld HSI devices have been proposed. Neittaanmäki-Perttu et al.^46^ used a prototype handheld HSI camera, based on a Fapry–Perot interferometer (FPI), which can acquire data in a snapshot-like manner. The device was positioned at a short distance from the skin and illumination was provided by a halogen lamp with fiber optic ring. Fabelo et al.^62^ also used a handheld prototype, with a snapshot HSI camera at its core. The system captures a $12\times 12\;{\mathrm{mm}}^{2}$ area with a small spatial resolution (50×50  pixels) in less than a second. They used a similar halogen illumination system with a fiber optic ring guide. The device is attached to the skin surface by a dermoscopic contact structure. Reports by Prigent et al.^34^ and Borisova et al.^29^ did not provide any information about imaging design, spatial resolution, or captured area.

### 3.3 Multispectral Prototype Systems

Most MSI systems consisted either of a monochrome sensor and multicolor LED lights or of a tunable filter and a single light source. Li et al.^50^ used a 12-bit monochromatic camera, together with an infrared enhanced lens, halogen illumination, and a bandpass filter for wavelength selection. The filter targeted eight center wavelengths in the range 400 to 1000 nm (420, 542, 581, 601, 726, 800, 860, and 972 nm) with full-width at half-maximum 10 to 41 nm. Delpueyo et al.^58^ combined a monochromatic CCD sensor with 32 LEDs (of eight wavelengths in range 400 to 1000 nm) in a ring formation instead of a filter. The LED wavelengths (414, 447, 477, 524, 671, 735, 890, and 995 nm) were selected to match the absorbance characteristics of skin chromophores. Aloupogianni et al.^61^ used a similar approach, but with an RGB camera and seven LEDs in the range 400 to 700 nm (450, 465, 505, 525, 575, 605, and 630 nm), instead. They combined the response of each RGB channel under a specific LED light to construct nine-channel MSI. Li et al.^50^ used a diffuser to scatter the light, while Delpueyo et al.^58^ and Aloupogianni et al.^61^ installed crossed polarizers between the illumination source and the sensor.

### 3.4 Takeaways

Since HMSI is still a developing technology, there is great variability in the imaging equipment used in each eligible study, as shown in Tables 2 and 3. The bulk of the reports used either general HMSI cameras or research prototypes. All acquisition systems captured information in at least the VIS range. Some systems acquired information up to the NIR range. The maximum spectral resolution for HSI was ∼0.6  nm and the maximum number of channels was 10 for MSI and 1127 for HSI. For applications in gross pathology of the skin, experimentation started with MSI prototypes and gradually expanded to HSI. The initial trend for melanoma versus nevus classification was to target specific wavelengths that coincide with critical points of the absorbance curves in Fig. 1. However, for a larger capture area or a variety of pathologies using the entire HSI spectrum is more appropriate.

Most studies used immobile HMSI systems. Only a few studies reported on the capture area, spatial resolution, or imaging speed. Capture duration ranged from seconds to minutes. Regarding applications on *in vivo* tissue, breathing or unconscious movement should be taken considered. An immobile system with snapshot scanning is more appropriate to acquire accurate spectral signatures. While it is noted that a light-weight handheld device is easier to use in a clinical setting, this should be limited to small (≤10  mm) capture areas. In addition, fast capture time is preferable to avoid data noise due to movement. Systems that try to emulate the function of a dermoscope are attached to the skin surface or positioned a few mm away. However, most systems were positioned at a distance of a few cm away from the target. This affects the spatial resolution and the maximum area that can be imaged. In this regard, a larger distance is preferable, but a macroscopic lens can be used to improve spatial resolution. Another parameter is the illumination condition. Studies were split in half between those that used LED lamps and those that used halogen lamps. Fiber-optic lighting is also an option. The use of polarizers helps to reduce noise and saturation, therefore is suggested. In addition, a dark box for measurements should be considered, to avoid the influence of ambient light.

Based on the above considerations, the following HMSI systems show the most promise. The system proposed by Suárez et al.^41^ is suitable for capturing a large area, which can facilitate the diagnosis of multiple types of lesions. It is capable of depth measurements, due to the high penetration of wavelengths in the NIR range. For lesions small in size and a dermoscope-like function, one can build a system similar to the one proposed by Fabelo et al.^62^ or Neittaanmäki-Perttu et al.^46^ Portable handheld devices can be easily incorporated in clinical practice. The fast acquisition time in a snapshot manner minimizes noise due to patient movement.

## 4 Preprocessing Schemes

**Fig. 5:**
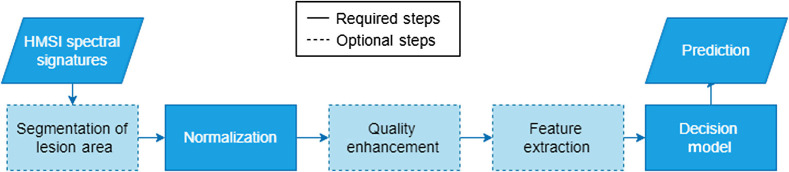
Flowchart of a typical processing scheme for HMSI spectral signatures. Dashed lines indicate optional steps, while full lines indicate required steps.

Due to the complex nature of HMSI information, some form of preprocessing is applied before feeding them to the decision model. A common flowchart from data acquisition to final prediction is provided in Fig. 5. Regardless of the imaging acquisition system, some form of normalization is necessary to effectively compare spectral signatures of different origins. Additional processing is sometimes applied to increase the quality of spectral signatures and to remove noise. Alternatively, HSI data can be estimated and reconstructed from MSI to increase information detail without using HSI equipment. Furthermore, large feature vectors, such as HSI signals, suffer from the curse of dimensionality. According to Hughes phenomenon, as dimensions increase from multi- to hyperspectral, there is some critical band number above which classification performance starts to decrease.^68^ Inherent redundancies in HSI hinder classification and generalization. Moreover, a larger feature space requires an exponentially larger training set. Consequently, feature extraction and feature selection have been proposed as a preprocessing step that reduces dimensionality. Some of the acquisition systems above^69^ offer software tools for preprocessing or automated analysis of the spectral information. However, most studies employed custom schemes for data processing, built in MATLAB or Python. In this section, we describe previously investigated preprocessing schemes for HMSI data.

### 4.1 Normalization

Normalization of spectra can refer to minimizing bias from nonuniform spatial illumination, different tissue type, or different patient. Measurement normalization greatly affects the performance, especially when machine learning is used. Moreover, it is necessary in order to convert HMSI measurements into reflectance ratios. The necessity of interpatient normalization depends on the task. For example, it is required for training an on-the-fly system of intraoperative margin detection, using previous samples of the same patient. However, it might not be necessary for a coarse classifier used in broader applications. For a large capture area, the spatial distribution of illumination intensity becomes inhomogeneous and nontrivial. Since the optical properties of tissue are affected by water content and temperature,^70^ additional corrections are required. For this reason, it is essential to control experimental conditions and calibrate the captured signals.

**Fig. 6:**
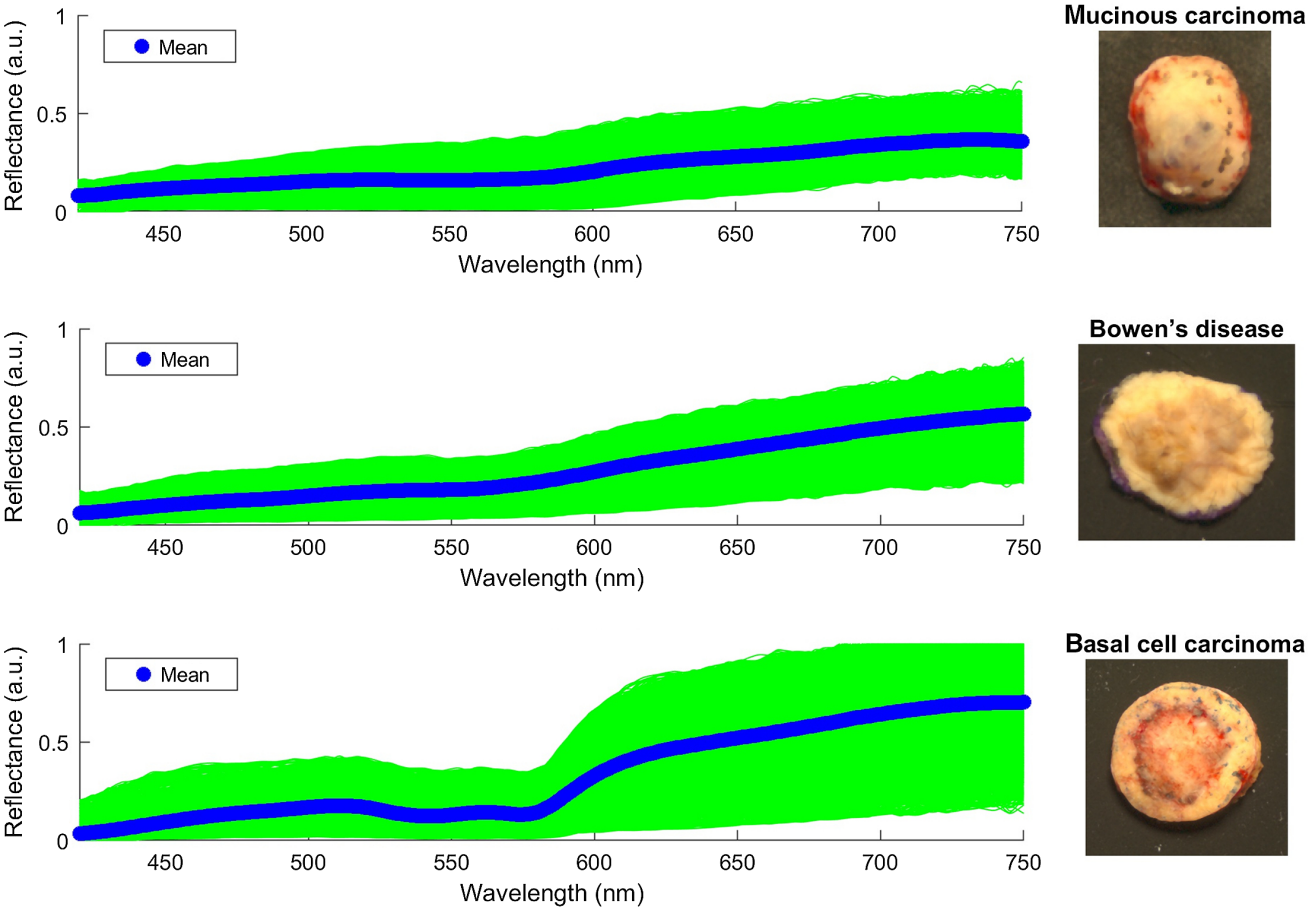
*Ex vivo* spectra for PSL, adapted from unpublished work by Ref. 71. The blue line represents the average. Depending on the tissue type, spectral signatures of each pixel differ in shape and value. Spectral signatures for mucinous carcinoma and Bowen’s disease are smoothly rising lines, whereas for BCC there is a sudden slope around 570 nm, consistent with the local maximum of hemoglobin absorption. Reproduced with permission.

The most popular approach for biological spectra is min-max scaling, using a dark current image and a white reference image.^39^^,^^45^^,^^52^^,^^55^^,^^62^ The reflectance spectrum r then becomes

$$r_{\mathrm{norm}}(x,y,\lambda )=\frac{I(x,y,\lambda )-B(x,y,\lambda )}{I_{0}(x,y,\lambda )-B(x,y,\lambda )},$$ (6)

where I is the raw spectrum, $I_{0}$ is the spectrum of the white reference object, and B is the dark current signal. An example of min-max scaled spectra is shown in Fig. 6. Delpueyo et al.^58^ multiplied the min-max scaled HSI with a the reflectance spectrum of the white reference. An alternative approach is that of optical density (OD), an expression of absorbance, used in Refs. 30,37,38,43,59. OD at each image pixel is defined as

$$\mathrm{OD}(\lambda )=-\log\;\frac{I(\lambda )}{I_{0}(\lambda )},$$ (7)

where log is the decimal logarithm, I(λ) is the intensity of the tissue-reflected light, and $I_{0}(\lambda )$ is the reflected light by a reference white object (usually with 99% reflection and minimal absorption). Delpueyo et al.^58^ calculated absorbance more straightforward, as minus the logarithm of normalized reflectance. Scaling takes into account the sensitivities of the sensor (due to the use of the black image), while OD considers the influence of sensitivity discrepancies as irrelevant. Pardo et al.^59^ applied additional normalization with normal standard variant to normalize interpatient variability of captured spectra. Each reflectance measurement x belonging to a patient is scaled by subtracting the mean μ and then dividing by the variance σ of that group. This way, bias and trend are removed from reflectance measurements.

### 4.2 Quality Enhancement

Noise is omnipresent in all measurements, but is exacerbated in HSI due to noise induced by patient movement (during *in situ* measurement), nonuniformity of illumination and illumination fluctuation in the long period that is required for capture. There are two options for denoising, in the form of smoothing in the spatial dimensions (used in Ref. 33) or smoothing in the spectral dimension (used in Refs. 62, 64). Errors in normalized values can be fixed by limiting them to range [0, 1]. Fabelo et al.^62^ and Leon et al.^64^ used a calibration scheme based on min-max scaling, followed by band-pass filtering of extreme bands, noise removal by smoothing the spectrum and another rescaling step to scale spectra in range [0, 1]. Commercial software, such as ImageJ, can be used to stabilize artifacts induced by patient motion.^37^

In cases of noise with great variability, more complex schemes can be employed. Li et al.^50^ developed an algorithm for the removal of outlier spectra, based on variance restriction. Outliers were present regardless of tissue type and performance increased 20% from the baseline model in terms of accuracy. However, it should be noted that both baseline and improved performance was low (<80%) and hyperparameters are empiric, requiring adjustments depending on the dataset and application.

### 4.3 Spectra Reconstruction

In cases of RGB or MSI systems, it can be hypothesized that useful information is missing due to the small number or selection of bands. For this reason, some studies attempted to reconstruct HSI spectral curves from a few RGB/MSI measurements. Assuming that MSI measurements g are

g=Hr+n, (8)

where H is the system parameter matrix (illumination and sensitivity), r is the reflectance spectrum, and n is the additive noise, we need to solve the ill-posed inverse problem to recover the HSI r spectrum. Delpueyo et al.^58^ calculated simply the HSI spectrum (59 points) as a spline interpolation by the measured reflectance values (eight channels). Kim et al.^55^ reconstructed HSI spectra (33 points) from RGB (three channels) by calculating a conversion matrix based on a collection of reference RGB and HSI data. The conversion matrix for reconstruction was learned by second-order multivariate polynomial regression, while the influence of additive noise was ignored.^72^ Aloupogianni et al.^61^ performed a reconstruction from MSI (seven channels) to HSI (81 points) based on Wiener Estimation with spatial denoising by Bayesian Interference.^73^ Reflectance is estimated using a smoothing matrix of the autocorrelation of reference spectra and a matrix of noise covariance.

### 4.4 Feature Selection

Feature selection maintains the original values of features, hence is necessary when model interpretability is important. During development stages for HMSI systems, spectral bands of interest are selected empirically. Quinzán et al.^47^ and Liu et al.^45^ aimed to create an appropriate filter bank for a targeted pathology, therefore, used supervised sequential floating forward selection, with a distance metric. Liu et al.^45^ performed a stability analysis that showed that modifications in the center wavelength of selected filters, affected performance considerably, and a shift should be limited to 4 nm. Kato et al.^66^ proposed two manually selected channel sets, namely [530, 540, 590] nm and [500, 620, 740] nm, with the former performing better.

### 4.5 Feature Extraction

Feature extraction is used to transform the dataset into a subspace that is more appropriate for classification and segmentation tasks. The number of transformed components can be limited, effectively reducing the dimensions of transformed samples. The most common unsupervised method for dimension reduction is principal component analysis (PCA) and similar variants, such as singular value decomposition (SVD) and Karhunen–Loeve (KL) transform. The goal of PCA is to transform the dataset in a new subspace so sample variance is maximized, under constraints of orthogonality. At the same time, PCA denoises the dataset, under the assumption that noise has low variance. Pardo et al.^59^ used KL in the form of Sequential KL (SKL) to transform the dataset while preserving information fidelity. SKL calculates SVD efficiently and with a dynamic threshold for dimension reduction. The training was done using manually selected, square regions of interest (ROI), that do not include border or fringe regions. Randeberg et al.^33^ used a variation of PCA called minimum noise fraction transform (MNFT), which performs well on signal-dependent noise.^74^ Forward and inverse MNFT was used for denoising, and then MNFT was applied again for feature extraction purposes. Prigent et al.^34^ proposed the use of projection pursuit (PP) to reduce dimensions before classification. PP searches for non-Gaussian projections in a lower dimension but is computationally expensive. Independent component analysis (ICA) is an alternative unsupervised technique that assumes independent components with non-Gaussian distributions, instead of principal components. ICA can be implemented with FastICA^33^ or JADE^34^ for a small number of components. Neittaanmäki-Perttu et al.^46^^,^^49^^,^^54^^,^^60^ used vertex component analysis (VCA),^75^ an unsupervised linear unmixing technique that detects a predefined number of pure components from the spectral signature. The coefficients of pure components were presented in the form of ”abundance” or concentration maps, in which patterns are unmixed further using filter vector algorithm (FVA). These maps served as input to classification.

The techniques above try to solve a problem of blind source separation because they assume no or minimal knowledge about the components that comprise the HMSI signal. However, other techniques can be trained on labeled data, if available. In many instances, linear discriminant analysis (LDA) is more powerful than PCA, because it maximizes interclass variance while minimizing intraclass variance. However, LDA is supervised and required prior class labels. Liu et al.^45^ used PCA to reduce HSI data to a 28-point space, before additional reduction with multiclass LDA. Nishino et al.^44^ employed a flavor of multiclass LDA that is based on canonical coordinates and canonical discriminant analysis (CDA) for NIR spectra.

Apart from spectral features, HMSI systems provide also texture and color features. To make use of spectrospatial information, Aloupogianni et al.^61^ applied a multispectral multiscale local binary pattern operator that extracts texture characteristics from slices of the HSI data cube. Incorporation of texture information in the classification showed better performance compared with the case of using standalone spectral information. Delpueyo et al.^58^ used as features the color coordinates in CIELAB space and color differences ΔE. On top of the extracted feature vector, they calculated texture features in an image segment, based on first- and second-order statistics, including mean, variance, entropy, energy, and third central moment. Lorencs et al.^53^ also extracted measurement statistics as features. Biological indexes that describe chromophore concentrations have also been proposed, such as Erythema index E=I(660)/I(545)^58^^,^^69^ and Bilirubin index B=I(450)/I(660).^58^ In an unconventional approach, Zheludev et al.^51^ first increased the dimension of input vectors via Framelet transform, selected some dozens of features, and then reduced dimension with diffusion maps.

### 4.6 Takeaways

Various preprocessing steps can be used sequentially for HMSI analysis. Dataset-wide normalization using a fully reflective and a dark reference is an essential step, especially if complex models are used later. Quality enhancement with average filtering should be used (if used at all) with caution, because it may erase spectral features in HMSI data with low spatial resolution. Dimension reduction is optional, while it depends on the size of the dataset. Feature extraction or selection can alleviate ill-posed problems with a large input vector compared with the available number of data samples. However, it is an optional step and if used, methods with different assumptions about the data should be compared. PCA and VCA are suitable as a base technique. In addition, feature extraction is not recommended when a complex decision model is used, to avoid overfitting. In studies where texture information was incorporated in segmentation, it assisted performance.

## 5 Classification and Segmentation

**Fig. 7:**
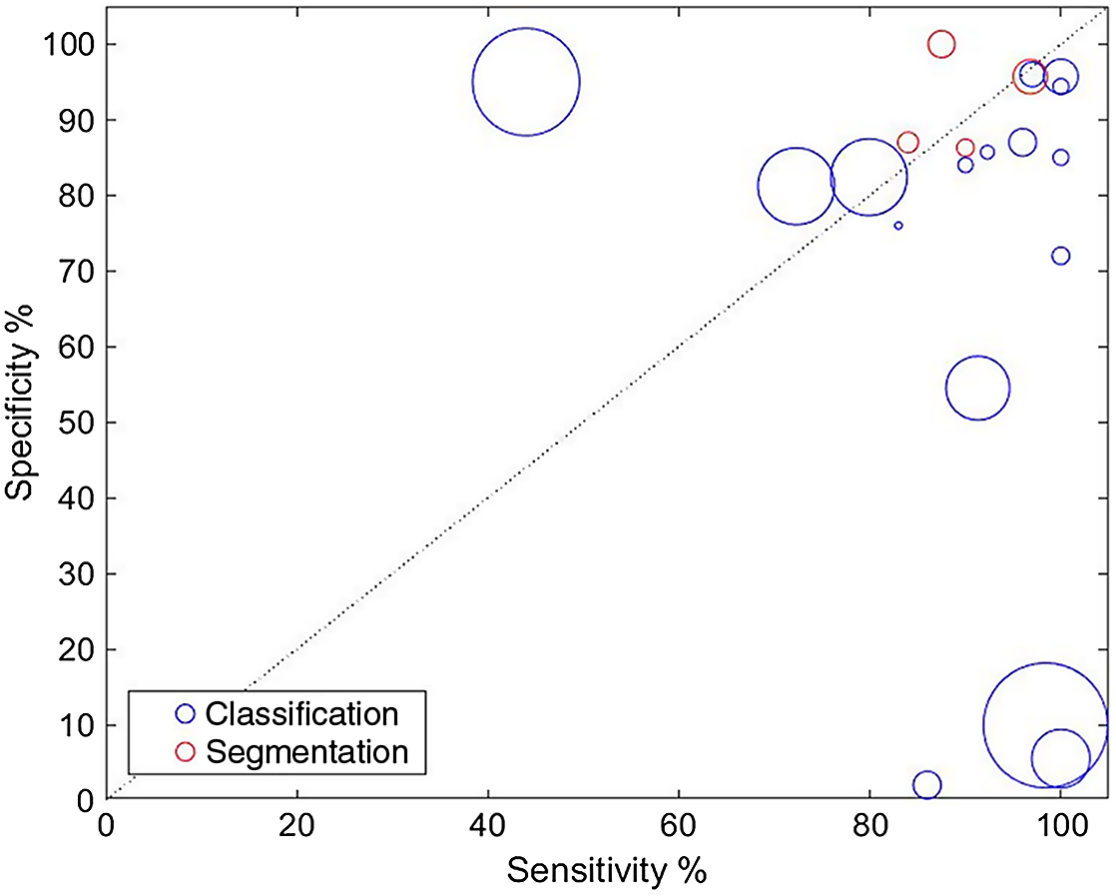
Sensitivity against specificity for eligible studies. The size of the marker is proportional to the number of lesions included in the study.

In this section, we summarize techniques for classification and segmentation, depending on the type of tissue and target lesions. A full list of methods and performance is provided in Table 4. In studies where lesion number was not reported, lesion number was assumed as equal to the total number of patients. Studies that did not report sensitivity or specificity were not included. The performance of various studies in terms of sensitivity and specificity is demonstrated in Fig. 7. Studies with large datasets showed skewed performance, either toward good specificity with low specificity or vice-versa. These specific studies refer to commercial systems^35^^,^^36^^,^^56^ that did not provide a detailed explanation of the classification decision making. Apart from Ref. 58 that showed high sensitivity with low specificity, the rest of studies reported a balanced performance of sensitivity and specificity, with both above 80%. Studies that reported accuracy instead of sensitivity and specificity had generally poor performance, ranging from 75% and above.

**Table 4:** Performance of eligible studies.

Ref.	Method	Scale	Acc.	Sens.	Spec.	Task
33	MNFT+ICA+SAM	Pixel	N/A	N/A	N/A	seg.
34	PP+ICA	Pixel	100.0%	N/A	N/A	seg.
35	Scoring algorithm	Image	N/A	44.0%	95.0%	clf.
36	MSDSLA	Image	N/A	98.4%	9.9%	clf.
37	Discrimination index	Patch	N/A	86.0%	2.0%	clf.
38	Discrimination index	Patch	N/A	N/A	N/A	clf.
39	Discrimination index	Image	N/A	90.0%	84.0%	clf.
40	Discrimination index	Image	N/A	100.0%	94.4%	clf.
41	SMOTE+SVM	ROI	88.5%	100.0%	85.0%	clf.
42	Discrimination index	Image	N/A	92.3%	85.7%	clf.
43	Discrimination index	ROI	N/A	N/A	N/A	clf.
44	CDA	ROI	90.2%	N/A	N/A	clf.
45	PCA+QDC	Pixel	N/A	97.0%	96.0%	clf.
46	Multilayer perceptron	Pixel	N/A	100.0%	N/A	seg.
47	SMOTE+SVM	ROI	N/A	100.0%	72.0%	clf.
48	Discrimination index	Image	N/A	96.0%	87.0%	clf.
49	VCA+FVA	Pixel	94.7%	N/A	N/A	seg.
50	SVM+outlier detection	Pixel	96.0%	N/A	N/A	clf.
20	MSDSLA	Image	N/A	83.0%	76.0%	clf.
51	Framelets+CaRT	Patch	N/A	N/A	N/A	seg.
52	Discrimination index	N/A	Pixel	N/A	N/A	seg.
30	Discrimination index+PCA	N/A	Pixel	84.0%	87.0%	seg.
53	Threshold classifier	Pixel	96.8%	100.0%	95.7%	clf.
54	VCA+FVA	Pixel	N/A	90.0%	86.3%	seg.
55	Reconstruction+statistics	Pixel	N/A	N/A	N/A	seg.
56	MSDSLA	Image	N/A	100.0%	5.5%	clf.
57	Meta-analysis	N/A	N/A	92.9%	43.6%	clf.
58	Importance+thresholding	Image	N/A	91.3%	54.5%	clf.
59	SVD+KDE+MAP	Pixel	96.0%	96.8%	95.7%	seg.
29	Reflectance average	N/A	N/A	N/A	N/A	clf.
60	VCA+FVA	Pixel	75.0%	N/A	N/A	seg.
61	RF+texture	Pixel	83.3%	N/A	N/A	seg.
62	SAM+genetic algorithm+SVM	Pixel	75.3%	N/A	N/A	clf.
63	ANN	Pixel	N/A	91.6%	N/A	clf.
64	Kmeans+SAM+SVM	Pixel	N/A	87.5%	100.0%	seg.
65	Input network+GoogleNet	Image	77.2%	72.3%	81.2%	clf.
66	GoogleNet+channel selection	Image	N/A	79.9%	82.4%	clf.

Note: N/A, not available; seg., segmentation; clf., classification. Other abbreviations are listed in the main text.

In the following section, a list of available classification/segmentation models and a short description is provided. In the next section, the systems with the best performance for each task are presented in detail. The section ends with key takeaways about decision making models.

Items are ordered according to publication year. Only studies that reported performance are included.

### 5.1 Overview of Proposed Methods

• Discrimination index is a value that can discriminate between classes. The calculation of the index depends on the target pathologies.^37^^,^^43^^,^^67^ A list of proposed indexes is provided in Table 5.
• Thresholding is a segmentation method that separates the pixels in a sample into two or more segments. The threshold value can be selected manually from a scatter plot or calculated adaptively. Thresholding is easy to apply after dimension reduction,^33^^,^^34^^,^^39^^,^^53^ calculation of a discrimination index,^30^^,^^37^ or other parameter.^58^^,^^59^
• K-means clustering (KC) is an unsupervised technique for data categorization in a fixed number of classes so that within-cluster variance is minimized. In Ref. 64, it was used for segmentation of smaller tissue regions, before supervised classification.
• K-nearest neighbors (KNN) is a nonparametric supervised classifier, which uses a distance metric to identify labeled data close to a sample, to classify the latter. KNN is simple but inefficient for larger datasets. KNN was compared against other classifiers in Ref. 61.
• Linear/quadratic discriminant classifier (LDC/QDC) is a supervised statistical classifier that identifies a surface that maximizes class separation. Class variances are assumed equal in LDC, while this is not necessarily true for QDC. The two where compared in Ref. 76 for multiclass classification, while Ref. 30 applied Fisher’s LDA in a “one-vs-rest” classification scheme.
• Artificial neural networks (ANN) are a class of supervised learning, which can be used to discriminate nonlinearly separable datasets. Multilayer perceptron, a primitive form of ANN, was used in Ref. 46, while a four-layer ANN was investigated in Refs. 63 and 64.
• Pretrained deep learning models include image classification and segmentation ANN networks, which were trained and optimized on exceptionally large datasets. Common pretrained networks are GoogleNet,^65^^,^^66^ ResNet, and VGG. Because they are trained on three-channel RGB images, conversion of the input structure or the input layer is necessary.
• Support vector machines (SVM) are a supervised classifier that looks for a hypersurface transformation that separates classes. It can be manipulated using *a priori* class frequencies, penalties, and different transformation kernels. Because of its effectiveness on high-dimensional datasets and ill-posed problems, it was used for classification in Refs. 50, 61, 62.
• Random forest (RF) is a supervised classifier, an ensemble of decision trees. It can achieve reasonable results with little training and was used in Refs. 61, 62, and 64.
• Spectral angle mapper (SAM) is a similarity measure specifically for HSI data, which groups samples according to a library of reflectance spectra. The selection of reference spectra can be achieved manually^62^ or using a purity index.^33^ It was proposed as a semiautomatic coarse classifier, to produce large quantities of labeled HSI pixels.^62^^,^^64^ SAM statics over an ROI were the building block of a proposed melanoma discrimination index.^39^^,^^48^
• Maximum *a posteriori* (MAP) estimation uses Bayes theorem on an assumed prior to estimate a posterior probability. MAP was used as a classifier of estimated conditional probabilities in Ref. 59.

**Table 5:** Summary of proposed discrimination indices.

Target	Index	Reference
Melanoma versus rest	p=OD(650)+OD(950)−OD(540)	37,67
Melanoma versus rest	$p=\frac{I_{545}}{I_{660}\cdot I_{940}}$	43
Melanoma versus rest	$\delta OD=OD_{\text{malformed}}-OD_{\text{healthy}}$	38
Melanoma versus PSL	D=Entropy(PDF(SAM(λ)))	39
Neoplasm versus rest	$WB(x,y{)}_{\lambda_{1},\lambda_{2}}=\frac{1}{\lambda_{2}-\lambda_{1}}\int_{\lambda_{2}}^{\lambda_{2}}OD(x,y,\lambda )d\lambda$	52
Malignancy versus rest	$D=WB{\left(x,y\right)}_{530,600}-WB{\left(x,y\right)}_{600,670}+OD(670)-OD(600)$	52

Note: OD(λ), optical density at wavelength λ; I(λ), spatial subimage at λ; PDF, probability density function. Other abbreviations are listed in the main text.

Despite the variety of proposed methods, they are defined by different assumptions, thus producing sometimes different results. SVM and RF are classifiers commonly used in medical applications with good classification results. SVM is effective in high-dimensional spaces and memory efficient, but may fail with a large dataset or noisy data. On the other hand, RF is robust to overfitting and with a few hyperparameters, but is slow in producing predictions real-time and does not focus on data description, making it hard to interpret. MAP produces fixed point estimates for predictions, which can be useful to denote confidence in the results. LDC produces impressive results assuming linear separation, but might underperform on uncommon data that do not follow normal distribution. KNN is a lazy model, which is easily affected by outliers and cannot be scaled effectively. ANN and pretrained models are preferred for segmentation problems, where the result is a 2D mask of class labels. However, they require large amounts of data samples. KC and SAM can be combined for segmentation problems and be applied on standalone image data.

### 5.2 High-Performing Systems

#### 5.2.1 Melanoma versus nevus

**Fig. 8:**
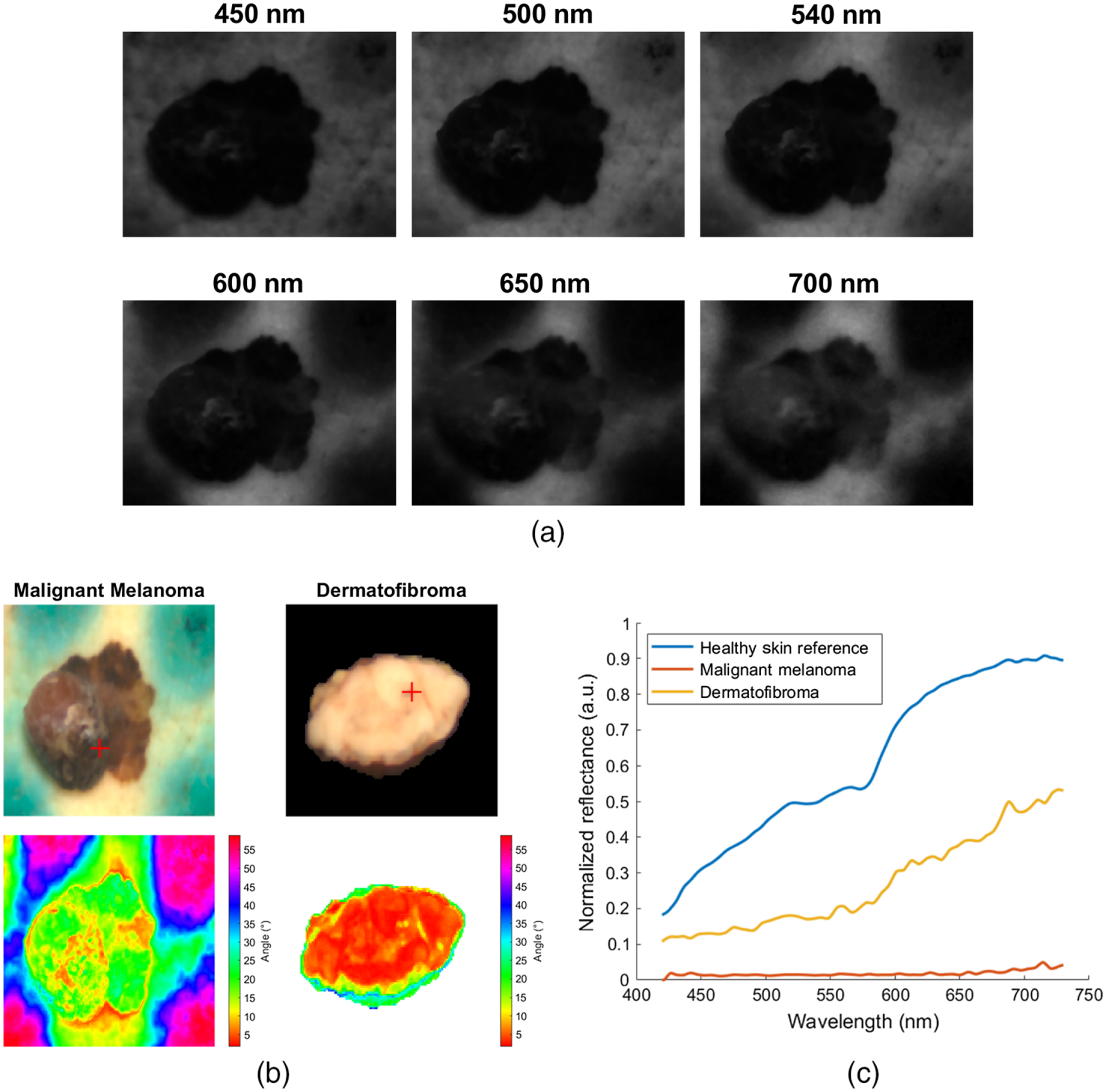
Example of initial processing for an MM. Spectral signatures from HSI subimages in (a) are used to calculate SAM values in (b). Pseudo-RGB images and SAM angles for MM (left) and benign dermatofibroma (right) are presented in (b). Spectral signatures of the highlighted red points in (b) are shown in comparison to the reference signature of healthy skin in (c). The angles of melanoma signatures are larger than those of dermatofibroma. The blue marks surrounding the melanoma tissue are pathology stains. Adapted from unpublished work by Ref. 71. Reproduced with permission.

A straightforward approach toward discriminating between melanoma and benign nevus is to calculate an HSI-based index or map, evaluate it visually, and apply a cutoff threshold. Nagaoka et al.^39^^,^^40^^,^^42^^,^^48^ took into account the unstructured nature of melanoma and calculated a discriminator index based on the entropy of a probability density function (PDF). An example of SAM angles for MM and benign dermatofibroma is shown in Fig. 8. They used SAM as a building block for the probability function. Afterward, they applied a threshold for discrimination between melanoma and other PSL. They modified the index by adding an upper limit to SAM frequencies, so melanoma false negatives are reduced. They found a possible correlation between this index and the existence of melanoma cell at the dermal/epidermal junction, which can be useful for cancer staging. Alternatively, another option is to calculate multiclass conditional probabilities of the preprocessed spectral signatures. Pardo et al.^59^ calculated probabilities using multivariate kernel density estimation (KDE) with Silverman’s rule of thumb estimator. Classification of the reduced spectrum is done with MAP, given dynamically adjusted class weights. The three trained feature bases did not extract specific absorbance properties, but instead a cumulative response, focusing at the 550 nm (hemoglobin absorbance peak) and the red and near infrared region (melanin absorbance slope). Their proposed method achieves fast execution and good results under various cross-validation schemes, includes only one degree of freedom and can be optimized to eliminate false negatives.

#### 5.2.2 *In vivo* pigmented skin lesions

In this case, carefully selected indexes like in the previous case cannot perform well, due to the great variability of spectral signatures from different pathologies. Fabelo et al.^62^ and Leon et al.^64^ both prepared two-stage classification systems. In the first stage, the lesion area was segmented. In the second stage, a carefully tuned SVM classifier produced class predictions. Hyperparameters of the SVM can optimized with a genetic algorithm, while a radial basis function kernel is preferred. However, such a model may suffer from low sensitivity (large number of false negatives).

#### 5.2.3 *Ex vivo* pigmented skin lesions

Aloupogianni et al.^61^ achieved testing accuracy >80% with an RF classifier trained on a feature vector consisting of spectral and textural features. They suggested that incorporation of textural features assists classification. In addition, augmentation of the dataset with the inclusion of tissue samples after formalin fixing improved classification performance. To visualize disease margins, they first segmented RO) with region growing and then assigned probabilities of malignancy to each ROI according to the classification probabilities of the classifier.

#### 5.2.4 Other skin conditions

For the problem of inflammation detection, Kim et al.^55^ used reconstructed HSI from MSI to create maps of hemoglobin content, to detect carcinogenesis. They concluded their system is able to display concentrations of chromophores accurately, therefore can help in skin diagnosis. Nishino et al.^44^ classified different types of allergic reactions using an NIR camera system. Classification was achieved by thresholding the extracted CDA components from the preprocessing stage. Furthermore, they converted VIS spectra to L*a*b* channel to associate classification results with oxyhemoglobin content in the tissue.

#### 5.2.5 Injury classification

Liu et al.^45^ classified skin conditions of diabetic foot against healthy skin. After feature extraction, the preprocessed feature vectors were used in a QDC. They emphasized dimensionality reduction as a way to avoid overfitting to a small training dataset. While the results for ulcer versus healthy classification were promising, they noticed a drop in performance with the inclusion of a “rest” class of skin lesions.

#### 5.2.6 All lesions

Although HMSI-based commercial systems did not provide details about the classification process, they have been tested in large and diverse skin datasets. Multispectral digital skin lesion analysis (MSDSLA) using MelaFind displayed high sensitivity and acceptable specificity in a real-life clinical setting,^56^ as well as higher sensitivity than clinical and dermoscopy evaluations.^20^ However, there are considerable limitations in terms of banal lesions as well as the specificity of biopsy decision.^27^^,^^77^ Despite obtaining limited approval by the U.S. Food and Drug Administration in 2011, the tool was discontinued in 2017.^11^ SIAscope’s assistant software produces various views, among which melanoma, hemoglobin, and collagen view. Combined with a primary care scoring algorithm, it showed good discrimination results for melanoma,^35^ but nonmelanoma lesions were excluded from the dataset and sensitivity was low. Ferrante di Ruffano et al.^57^ performed a meta-analysis on 15 reports including commercial MSI computer-assisted diagnosis (CAD) systems and reported sensitivity as 92.9% (95% CI 83.7% to 97.1%) and specificity as 43.6% (95% CI 24.8% to 64.5%). They observed that MSI-based systems perform at least at the level of dermoscopy, the current golden standard in dermatology. However, current methods have been evaluated on heavily controlled datasets, including specified pathologies. The aforementioned commercial systems suffer from the drawback that the images produced by the software need to be evaluated by experienced dermatologists and are expected to replace the dermoscope in assisting diagnosis, not to provide a final diagnosis.

### 5.3 Takeaways

Many of the eligible studies emphasized feature extraction, resulting in simple visual evaluation of index images or semiautomatic thresholding. There were a few studies that used traditional machine learning classifiers and only five that used deep learning. For clearly identified tasks such as melanoma versus nevus classification, the use of thresholding on a discrimination index seems sufficient.^48^ In this regard, an MSI with a few channels is enough. However, for a multiclass problem, a more complex approach is necessary. Staple classifiers such as SVM can provide good results.^59^^,^^64^ It should be noted that synthetic minority oversampling technique (SMOTE)^41^^,^^47^ can alleviate the problem of unbalanced training classes for an SVM model. Integration of concepts prepared for remote sensing HSI, such as SAM and endmembers, can improve performance. On the other hand, ANN systems generally perform poorly compared with simple methods.^65^^,^^66^ This could be attributed both to the relatively small size of the training dataset, the large number of training parameters, and the unsuccessful learning of rare features.

## 6 Discussion

### 6.1 Data Acquisition

All but one, the systems in this study focused on the VIS range of wavelengths. In comparison to the extinction coefficient graph in Fig. 1, indeed characteristics of the curves are included in the 400 to 800 nm range. With the advancement of HMSI cameras, snapshot cameras can replace line-scan cameras. The capture of a small ROI can last as little as a few seconds, minimizing discomfort to the patient. The size of captured images varied widely, from 50×50 to 1200×1400  pixels. Because of the variety in sensor equipment, spectral resolution varied as well. Therefore, any preprocessing and classification scheme should be adjusted to the specifics of the HMSI system that was used for acquisition.

### 6.2 Preprocessing Schemes

Normalization greatly affects the performance, especially when machine learning is used. OD as an expression of absorbance is becoming obsolete. Min-max scaled reflectance information is preferred instead. In some cases, noise filtering was applied. However, depending on the resolution, this is not generally recommended, since such filters might also reduce peaks in the spectrum that do not represent noise. In addition, in the case of biological spectra, noise affects the location of a peak instead of the height of the peak. Therefore, that peak shift might disappear with spectral filtering. However, it might be beneficial to reduce artifacts around hair follicles,^33^ which contain high melanin content.

Relevant studies included a variety of feature extraction and feature selection methods. During the review process, it was evident that preprocessing techniques were selected “as is,” with empirical hyperparameter selection and no comparison to alternative schemes. For example, PCA and ICA have different assumptions about a component’s contribution to the total variance or intercomponent independence. Furthermore, apart from general methods, there are available dimension reduction techniques specifically for the classification of biological HSI spectra.^78^ There is a need for comparative evaluation of feature extraction methods to identify which is more appropriate according to lesion type and the task at hand. Further research is needed on whether preprocessing induces overfitting of the result or discards valuable components. In addition, there is a concern on whether it is more appropriate for dimension reduction to be trained on a patient’s reference data (interpatient) or a database of patient data (intrapatient).

### 6.3 Classification and Segmentation

HMSI methods in this review performed well compared with dermoscopy, with many studies reporting sensitivity and specificity <80%. A study on 463 lesions (of which 30% were malignant) reported 80% sensitivity and 82.6% specificity when using dermoscopy.^79^ A meta-analysis reported a cumulative 90.1% sensitivity and 74.6% specificity of dermoscopy-based systems.^57^ Dermoscopy and histological biopsy, despite both being gold standards at different stages of diagnosis, evaluate different parts of the tissue.^80^ Dermoscopy evaluates the entire tissue area, including color and patterns. Histopathological biopsy evaluates vertical sections and the cell structure in depth but evaluates only a small percentage of the tumor. In this context, depending on the acquisition range, HMSI can combine the two, provide detailed color and texture information together along with some depth information.

However, the studies with exceptionally high specificity and sensitivity in this review were evaluated on small datasets with carefully selected target lesions, which might positively skew performance. Larger datasets that contained a variety of lesions showed worse performance. Moreover, the classification systems should be able to be updated and retrained on a larger dataset to incorporate newly accumulated knowledge. Some classification models were dependent on biological absorbance properties, while others were data-driven. There is a lack of studies that evaluate alternative methods simultaneously. To minimize the need for participation of the medical staff in the analysis process, it is essential to develop fully automated classification methodologies.

A point of concern in classification is robustness and interpretability. Liu et al.^45^ assessed the stability of the classification system using Monte Carlo analysis. Other studies did not perform robustness checks. The explainability of the results is also contentious. Some results associated important wavelength for classifications with characteristic wavelengths for skin chromophores. However, when applying classification on an HMSI patch, there is a need to investigate ROI that influenced the classification decision, apart from the spectral dimension. Methods such as Grad-CAM^81^ and LIME^82^ have been proposed to explain the results of machine learning models for RGB images. Using explainable visualizations of the classification system, it is easier to develop a robust system, as well as to convince medical staff of the system’s fidelity. None of the eligible studies reported concerns regarding the patients’ safety. In some instances, opinions among medical staff are divergent. Most studies did not mention the number of doctors that performed manual classification and labeling. Labeling, training, and performance are subject to change when the majority vote of multiple doctors is included during the data collection and evaluation stages.

### 6.4 Limitations and Obstacles

A crucial issue in the development of CAD systems is validation with real data. Due to the high workload of pathologists and the discrepancies between clinical protocol and CAD system development requirements, it is difficult to obtain fully labeled datasets. In addition, to properly validate a system, a balanced dataset of both healthy and malignant samples is preferred. However, it is not ethical to perform histological biopsies on every single patient. This deficit of healthy labeled data affects the training of the CAD system and might increase false negatives. SVM is claimed to perform well with unbalanced datasets and indeed performed best in studies in this review. Furthermore, in cases where histology slides are available, they need to be registered to the HMSI cube. Movement and deformation of the tissue complicate image registration. In addition, histology slides are prepared from cross-sections of the tissue, while gross-level HMSI captures the surface of the tissue.

To train the segmentation of tumor effectively, a large number of samples is required. This makes the use of each pixel as one data point the obvious choice. Training on pixelwise labels ignores spatial information but provides more samples for training, improving accuracy. However, pixelwise samples will contain inherent correlations, due to being extracted from the same lesion/patient. On the other hand, training on patch-wide labels utilizes both spatial and spectral information. Therefore, an even larger number of tissue samples is required for adequate training and validation of the diagnostic model. This limitation in acquiring large, labeled datasets is what is hindering the application of deep learning algorithms on HMSI of skin lesions. Recently, Halicek et al.^83^ applied deep learning for tumor margin detection of nonskin head and neck tissue samples with promising results. Active learning can be used to speed-up development amid a lack of labeled HMSI.^84^^,^^85^

While HMSI-based classifiers show potential for automatic detection of cancer margins, there is a long way to go until they are incorporated in the clinical practice. Fink et al.^24^ compared a variety of noninvasive imaging approaches for melanoma detection and found none was able to provide a definite and final diagnostic result. Indeed, in eligible reports, there were cases with unexpected false positives and false negatives. To be useful in practice, HMSI-based CAD systems need to include fidelity estimates and display deciding factors regarding the segmentation result they produced.

Based on the results of this review, in our future work, we will aim to compare different preprocessing and segmentation schemes with each other and a common baseline. We will consider data augmentation and transfer learning methods to investigate the application of deep learning. In addition, we aim to investigate analysis techniques that are specifically developed for HMSI images, instead of traditional learning techniques. Finally, we will try to adjust the training process and performance toward good interpretability and explainability of the segmentation model.

## 7 Conclusions

A variety of HMSI-based methodologies for cancer segmentation and margin detection of skin lesions have been proposed. Most studies applied simple image processing or machine learning, due to small training datasets. Methodologies have been evaluated on heavily curated datasets, with a major focus to melanoma detection. Evaluation on larger datasets, comparison of a variety of methodologies, and estimation of robustness to unusual lesions is necessary. The choice of preprocessing scheme greatly influences the performance of the classifier. Dimension reduction is required to avoid redundancies that are inherent in HSI systems. Incorporation of both spatial and spectral information shows potential. To use HMSI for tumor margin detection in practice, the focus of system evaluation should shift toward explainability of the decision-making process.
